# Supplementary material for: Predicting visual function by interpreting a neuronal wiring diagram
Source: Nature. 2024 Oct 2;634(8032):113–23. doi: 10.1038/s41586-024-07953-5 (PMC11446822; doi:10.1038/s41586-024-07953-5)
Supplement: Supplementary file 6 — CRF and ERF predictions for individual TmY4 and TmY9 cells. Analogous to Supplementary Data 3, but for TmY target types. Shown are the top four monosynaptic pathways, the strongest pathway passing through each of the top ten intermediary types (ranking from Extended Data Fig. 7), and the trisynaptic pathway Tm1–TmY–Dm3–TmY (see the section entitled Prediction of spatial normalization). [file 41586_2024_7953_MOESM6_ESM.zip › DataS4/TmY9qΓèÑ/720575940613363710.pdf]

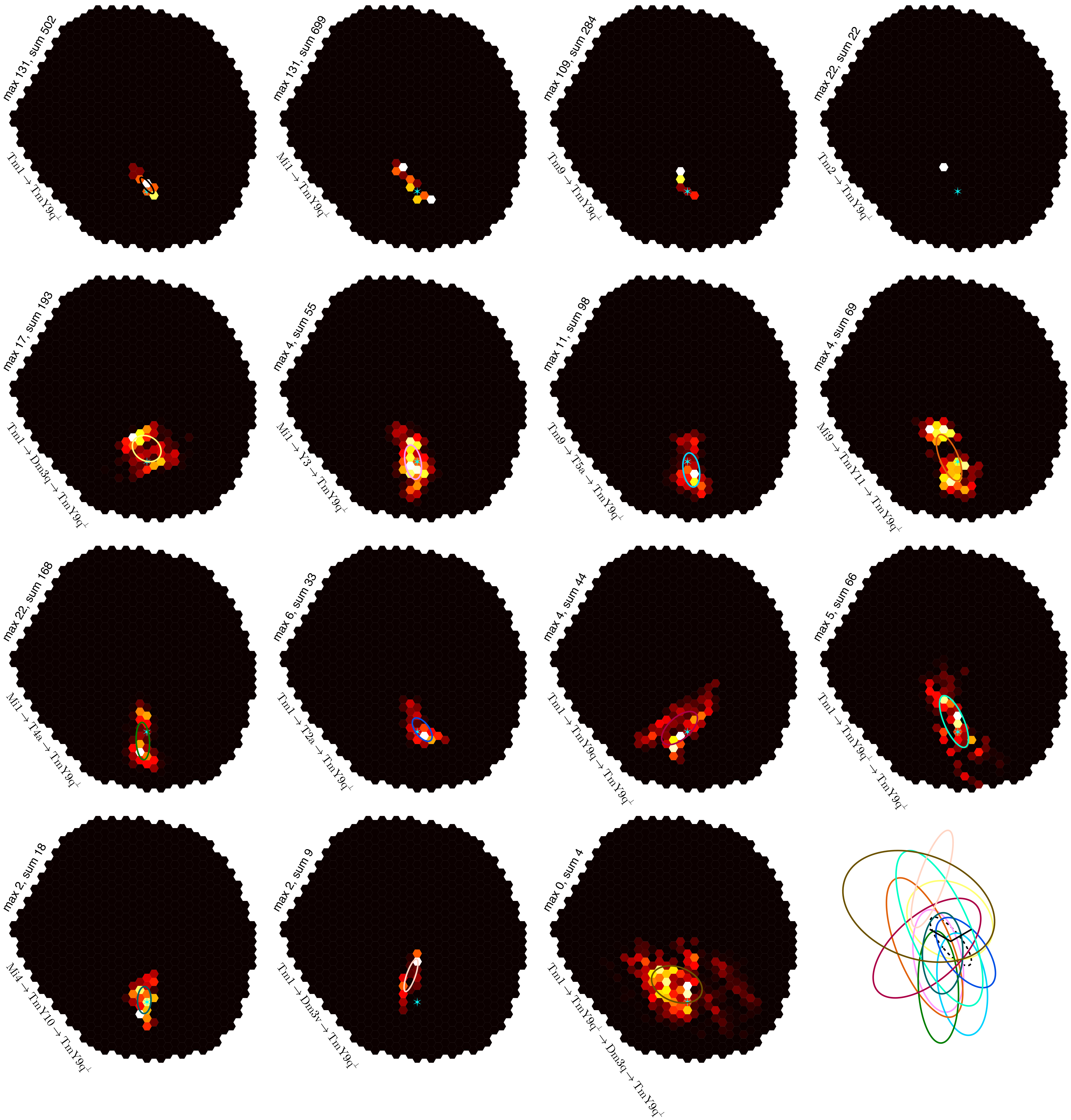

max 131, sum 502
Tm1 → TmY9q⊥
max 131, sum 699
Mi1 → TmY9q⊥
max 109, sum 284
Tm9 → TmY9q⊥
max 22, sum 22
Tm2 → TmY9q⊥
max 17, sum 193
Tm1 → Dm3q → TmY9q⊥
max 4, sum 55
Mi1 → Y3 → TmY9q⊥
max 11, sum 98
Tm9 → T5a → TmY9q⊥
max 4, sum 69
Mi9 → TmY11 → TmY9q⊥
max 22, sum 168
Mi1 → T4a → TmY9q⊥
max 6, sum 33
Tm1 → T2a → TmY9q⊥
max 4, sum 44
Tm1 → TmY9q⊥ → TmY9q⊥
max 5, sum 66
Tm1 → TmY9q⊥ → TmY9q⊥
max 2, sum 18
Mi4 → TmY10 → TmY9q⊥
max 2, sum 9
Tm1 → Dm3v → TmY9q⊥
max 0, sum 4
Tm1 → TmY9q⊥ → Dm3q → TmY9q⊥
